# A systematic review and meta-analysis of the prevalence of osteoarticular brucellosis

**DOI:** 10.1371/journal.pntd.0007112

**Published:** 2019-01-18

**Authors:** Shakirat A. Adetunji, Gilbert Ramirez, Margaret J. Foster, Angela M. Arenas-Gamboa

**Affiliations:** 1 Department of Veterinary Pathobiology, Texas A&M University, College Station, Texas, United States of America; 2 School of Public Health, Texas A&M University, College Station, Texas, United States of America; 3 Medical Sciences Library, Texas A&M University, College Station, Texas, United States of America; Oxford University Clinical Research Unit, VIET NAM

## Abstract

**Background:**

Infection of bones and joints remains one of the most commonly described complications of brucellosis in humans and is predominantly reported in all ages and sexes in high-risk regions, such as the Middle East, Asia, South and Central America, and Africa. We aimed to systematically review the literature and perform a meta-analysis to estimate the global prevalence of osteoarticular brucellosis (OAB).

**Methodology:**

Major bibliographic databases were searched using keywords and suitable combinations. All studies reporting the incidence and clinical manifestations of osteoarticular brucellosis in humans, and demonstrated by two or more diagnostic methods (bacteriological, molecular, serological, and/or radiographic) were included. Random model was used, and statistical significance was set at 0.05%

**Principal findings:**

A total of 56 studies met the inclusion criteria and were included in the systematic review and meta-analysis. There was an evidence of geographical variation in the prevalence of osteoarticular disease with estimates ranging from 27% in low-risk regions to 36% in high-risk regions. However, the difference was not significant. Thus, brucellosis patients have at least a 27% chance of developing osteoarticular disease.

**Conclusions:**

The prevalence of OAB is not dependent on the endemicity of brucellosis in a particular region. Hence, further research should investigate the potential mechanisms of OAB, as well as the influence of age, gender, and other socioeconomic factor variations in its global prevalence, as this may provide insight into associated exposure risks and management of the disease.

## Author summary

Brucellosis continues to be a global public health concern. It is caused by facultative, intracellular *Brucella* species. The most commonly described complication of brucellosis in humans is the infection of bones and joints, which is predominantly reported in all ages and sexes in high-risk regions, such as the Middle East, Asia, South and Central America, and Africa. In this current study, we systematically reviewed the literature and performed a meta-analysis to estimate the global prevalence of osteoarticular brucellosis. We demonstrated an evidence of geographical variation in the prevalence of osteoarticular brucellosis with estimates ranging from 27% in low-risk regions to 36% in high-risk regions. However, the difference was not significant. Therefore, the prevalence of osteoarticular brucellosis is not dependent on the endemicity of brucellosis in a particular region, and brucellosis patients have at least a 27% chance of developing osteoarticular disease.

## Introduction

Brucellosis is a neglected disease worldwide and a growing public health concern in high-risk countries. It is caused by facultative, intracellular *Brucella* species. *Brucella abortus* (cattle), *Brucella melitensis* (goats and sheep), *and Brucella suis* (pigs) are known to be the most pathogenic to their target hosts as well as humans [1–5].

Humans are considered incidental hosts of brucellosis, and can acquire the disease via various routes, including oral, conjunctival, respiratory, cutaneous, transplancental, blood, and rarely by bone marrow transplantation [1,2,6–8]. However, infection is typically by direct exposure to contaminated animal products (e.g. consumption of unpasteurized milk), genital secretions, aborted fetuses, infectious aerosols, and accidental vaccine inoculations [5–7,9–13].

In humans, brucellosis manifests as a non-specific, flu-like illness characterized by undulant fever, headache, myalgia, arthralgia, lymphadenopathy, hepatomegaly and splenomegaly, among others. The risk of adverse pregnancy outcomes has also been reported in pregnant women infected with *Brucella* species [1,14–18]. Although brucellosis causes minimal mortality, the severe debilitating morbidity associated with the disease is of negative socioeconomic impact due to the time lost by patients and care-givers from normal daily productive activities, and the detrimental effects of antibiotic resistance resulting from prolonged use of antibiotics for treatment of the disease [3,19–22].

Infection of bones and joints remains one of the most commonly described complications of brucellosis in humans [13,21,23–26], and is predominantly reported in all ages and sexes in high-risk regions, such as the Middle East, Asia, South and Central America, and Africa [27–38]. Frequently, *B*. *melitensis* is isolated in cases of osteoarticular brucellosis (OAB) in high-risk regions. However, in low-risk regions, such as the United States, *B*. *abortus* is the most commonly encountered *Brucella* species, followed by *B*.*suis* [13,32,39–43].

Osteoarticular brucellosis (OAB) can be acute, subacute, or chronic. It is often diagnosed because of complaints of pain in joints or an evidence of infection at one or more locations of the musculoskeletal system [29,44,45]. These symptoms can present as inflammation (such as swelling, pain, functional disability, heat, tenderness, and redness) of bone and/or joints, or radiological evidence of bone anomalies [24,29,44–46]. Osteoarticular involvement can occur at any time during brucellosis infection and the main sites of the musculoskeletal system that are affected include the joints, spine, extraspinal tissues, tendon sheaths, as well as muscles [13,45,47–49].

Generally, OAB presents as sacroiliitis, peripheral arthritis, spondylitis, and osteomyelitis. Sacroiliitis is the inflammation of one or both sacroiliac joints. The onset of sacroiliitis may be preceded by non-specific flu-like symptoms such as fever, chills, sweats, and malaise [50], and is associated with severe pain in affected individuals [13,29,32,51]. The associated severe and acute pain has led to several misdiagnoses of this condition as leg monoplegia, fracture of the neck of femur, and prolapsed intervertebral discs [13,29,32,52]. The incidence of sacroiliitis varies widely (about 2% to 45%) depending on *Brucella* endemicity of the reporting region (14). Peripheral arthritis is one of the most common complications associated with brucellosis [13,23,32,45,48], and may affect patients of any age [24,29,46,53]. Arthritis may present as monoarticular, oligoarticular, or polyarticular distribution accompanied by pain and swelling of the affected region, especially in acute conditions [28,29,46,52,54,55]. The incidence of *Brucella*-induced arthritis is about 3% to 77% (13,31,38). Large joints such as the knees and hip are the most frequently involved peripheral joints, and less commonly, ankles, shoulders, elbows, wrists, and sternoclavicular joints are affected as well [23,32,45,46,48,56–59]. Clinical presentations of *Brucella*-induced arthritis are not specific, and should be differentiated from other types of arthritis by clinical history and a positive blood or synovial fluid culture of *Brucella* in infected individuals. *Brucella*-induced spondylitis is an inflammation of the spine and large joints that causes more serious complications than arthritis [54,60–63], and it typically begins at the disco-vertebral junction, but may spread to the whole vertebrae and to adjacent vertebral bodies [13]. The most commonly affected region is the lumbar spine, especially at the L4 and L5 levels. Other sites affected are the thoracic and cervical spine [26,54,62,63]. The diffuse form of spondylitis covers the entire vertebral body, and may extend to the adjacent disc, vertebrae and epidural space [51,64]. Destructive brucellar lesions of the spine are commonly reported in adults and can occur in any spinal region at single or multiple levels [13,30,32,65–68]. Apart from serology and culture, clinical history is valuable in the diagnosis of spondylitis since the presenting features are similar to other causes of spinal disease such as tuberculosis (13). *Brucella*-induced osteomyelitis is an infection of bone resulting in its inflammatory destruction and necrosis. It presents as motor weakness or paralysis and has been associated with a high rate of therapeutic failure and functional sequelae [69].

Several clinical reports suggest that individuals with *Brucella* infection commonly present with osteoarticular complication. Moreover, the prevalence of OAB is variably reported (2%-77%), depending on the virulence of *Brucella* species involved, age group and sex of the individuals affected, diagnostic methods, and endemicity of the reporting region [21,36,45,48,59,60,67,70,71].

Until this study, no attempt has been made to integrate all published studies and reports to derive a robust prevalence estimate of OAB. Therefore, the objective of this report was to systematically review the literature and perform a meta-analysis to estimate a well-grounded prevalence of OAB, which will help to establish disease awareness, facilitate early detection of the pathogen, facilitate development and validation of diagnostic tests, as well as demonstrate the need for vaccine development for prevention and control.

## Methods

### Eligibility criteria

All studies reporting the incidence and clinical manifestations of osteoarticular brucellosis in humans, or where prevalence of the disease could be calculated from available data were included in this current study. Studies reporting infection of the bones and/or joints, demonstrated by two or more diagnostic methods (bacteriological, molecular, serological, and/or radiographic) were included. Studies involving co-infection with other pathogens, evaluating therapeutic or surgical responses in osteoarticular brucellosis patients, as well as animal experimentations were excluded. Furthermore, review articles, case-control studies, conference proceedings, and book chapters were excluded.

### Search strategy

Six databases were searched on March 6, 2018: Medline (Ovid), Global Health (Ovid), Northern Light Life Sciences (Ovid), CINAHL (Ebsco), Agricola (Ebsco), and Embase (Ovid). The searches included 3 concepts: brucellosis, prevalence or epidemiologic studies, and bone and joint infections or common manifestations of osteoarticular brucellosis such as arthritis, osteomyelitis, spondylitis, and sacroiliitis. (See S1 Text: Supplementary File for the details of the Medline (Ovid) search). The search was restricted to English Language reports and not restricted by year. In addition, references from the brucellosis entry from the Global Infectious Disease and Epidemiology Network (GIDEON) were collected. Cited and citing references of included and related reviews were retrieved using Scopus.

### Screening

Citations were uploaded to Rayyan, an application designed for sorting citations [72]. Titles and abstract were screened. Those that seemed relevant were added to RefWorks and the full-text were reviewed.

### Data extraction

Equivalent information was extracted from all included studies. This information comprised of the geographical region, sample size infected with brucellosis as well as those with osteoarticular involvement, age, sex, type of joints affected, and diagnostic methods (such as inflammatory signs, bacteriological culture, immunoassays, and radiographic imaging techniques). Prevalence and 95% confidence interval were calculated or extracted from the reported data.

### Data analysis

The prevalence estimates for osteoarticular brucellosis in this review were based on the total number of individuals with confirmed brucellosis (denominator) and a proportion of these individuals with one or more osteoarticular disease manifestations. The meta-analytic integration of the individual study prevalence estimate was carried out using Stata15 and its “metaprop” and “galbr” commands. The “metaprop” command was developed specifically for meta-analysis of proportions and is based on the Freeman-Tukey double arcsine transformation for stabilizing variances. The “galbr” command produces a graphical display of the amount of heterogeneity among studies included in a meta-analysis. The “metaprop” command uses the numerator and denominator and carries out the Freeman-Tukey double arcsine transformation and then applied as fixed and/or random effects models using inverse variance weighting. The numerator and denominator data were used to estimate prevalence and these data were transformed into the Freeman-Tukey double arcsine equivalent with standard errors using Excel, and the data were then used to generate the galbraith plots.

## Results

### Study search

**Fig 1:**
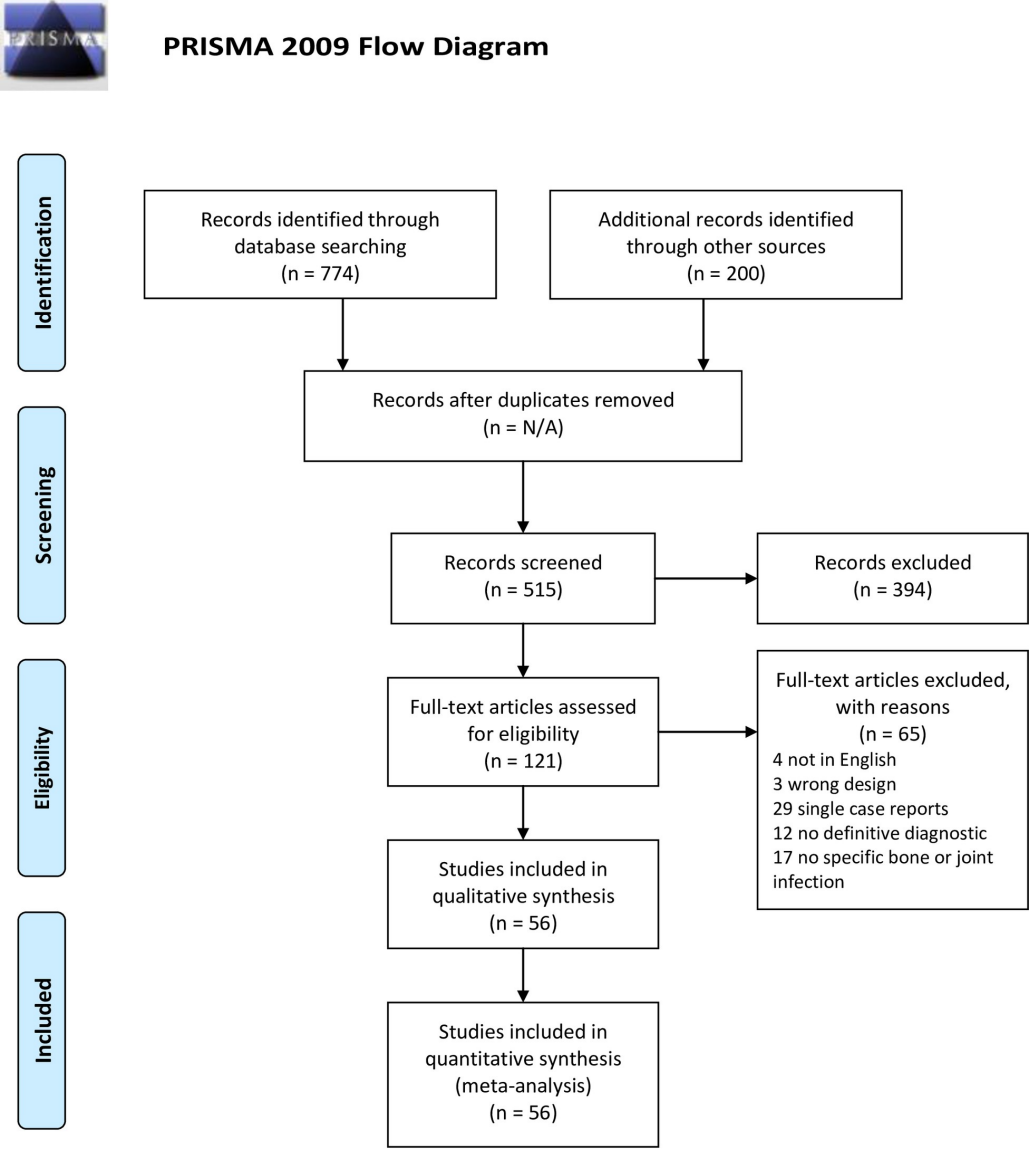
Flow-chart of systematic review of osteoarticular brucellosis.

A total of 974 publications were identified, which led to 515 articles being analyzed for full-text review. After full-text review, 56 published studies met the inclusion criteria and were used in the meta-analysis. Fig 1 details the process of article screening and selection following the Preferred Reporting Items for Systematic Review and Meta-Analyses (PRISMA) statement guidelines [73].

### Included studies

All articles included in this study were either prospective (32%) or retrospective (64%), and the authors reported acute or chronic cases of human brucellosis and associated complications. Most of the included studies were from the Middle East, especially Turkey (37.5%), Iran (16%), Saudi Arabia (9%), Israel (3.5%), Kuwait (3.5%), Jordan (1.8%), and Iraq (1.8%). In Europe, studies were also reported from Spain (9%), Macedonia (7%), Germany (1.8%), Portugal (1.8%), and Kosovo (1.8%). Only one report was from South America, specifically Peru (1.8%). The most represented countries were Turkey, Iran, Spain, and Saudi Arabia, respectively, and *B*. *melitensis* was the predominant species isolated from either blood or bone marrow cultures of infected individuals. The age range of the study population was 0–88 years old. 25% of the included studies reported childhood OAB while 8% reported OAB in adults. Most studies reported varying proportion of osteoarticular brucellosis in both males and females. Table 1 details the characteristics of all the studies included in this review.

**Table 1:** Characteristics of included studies.

Study	Country	Age	Sex ratio-Osteoarticular brucellosis (Male/Female)	Sample size (brucellosis)	Sample size (Osteoarticular brucellosis)	Prevalence/Proportion	Joints affected
Aktug-Demir et al., 2014	Turkey	18+	N/A	227	75	0.33	Sacroiliac, spine
Al-Eissa et al., 1990	Saudi Arabia	0–14	15/25 (37.5/62.5%)	102	40	0.39	N/A
Ariza et al., 1993	Spain	7–83	42/20 (67.7/32.2%)	530	62	0.12	Spine, hip, bursa
Aydoslu et al., 2006	Turkey	17–76	N/A	47	14	0.3	Sacroiliac, spine, peripheral arthritis
Benjamin et al., 1992	Saudi Arabia	N/A	N/A	157	57	0.36	Hip, knee
Benjamin and Khan, 1994	Saudi Arabia	0–12	N/A	190	70	0.37	Sacroiliac, spine, hip, knee, ankle, shoulder
Besharat et al., 2010	Iran		N/A	140	56	0.4	N/A
Bosilkovski et al., 2004a	Macedonia	3–78	N/A	331	196	0.59	Sacroiliac, spine, hip, bursa, sternochondral, costochondral
Bosilkovski et al., 2004b	Macedonia	4–69	18/15 (54.5/45.5%)	263	162	0.62	Hip
Bosilkovski et al., 2010	Macedonia	1–82	N/A	550	299	0.54	Peripheral arthritis, sacroilitis, spondylitis
Bosilkovski et al., 2013	Macedonia	0–14	N/A	317	133	0.42	Sacroiliac, hip, knees, ankle, bursa, shoulder, elbow, wrist, interphalangeal, sternoclavicular
Bukharie, 2009	Saudi Arabia	13–81	N/A	84	54	0.64	Spine
Bulut et al., 2011	Turkey	15–83	N/A	324	84	0.26	Sacroiliac, spine
Cirakli et al., 2015	Turkey	2–17	42/10 (80.8/19.2%)	52	11	0.21	Hip, knee
Colmenero et al., 1991	Spain	14–73	N/A	263	65	0.25	Sacroiliac, spine, ankle, olecranon bursa
Colmenero et al., 1992	Spain	14–82	N/A	593	58	0.1	Spine
Colmenero et al., 1996	Spain	14+	2/-	530	2	0.004	Sacroiliac, spine
Colmenero et al., 2008	Spain	>14	69/27 (72/28%)	918	96	0.11	Vertebral osteomyelitis
Dabbagh and Rasool 2009	Iraq	<10 >60	N/A	80	45	0.56	Knee, spine, sacroiliac
Dahouk et al., 2005	Germany	4–72	14/16	62	11	0.37	Sacroiliac, sternoclavicular, spine, bursa
Demiroglu et al., 2007	Turkey	15–79	N/A	151	51	0.34	Spine, sacroiliac, tendon
Ebrahimpour et al., 2017	Iran	15–80	299/165 (64.4/35.6%)	1252	464	0.37	Sacroiliac, hip, knee, ankle, elbow, shoulder
Eini et al., 2012	Iran	9–88	N/A	230	118	0.51	Spine
Fanni et al., 2013	Iran	2–14	N/A	34	26	0.77	Hip, knee, elbow, wrist, ankle, sacroiliac
Fruchtman et al., 2015	Israel	0–19	N/A	252	92	0.37	N/A
Gonen et al., 2013	Turkey	15–88	N/A	201	50	0.25	Sacroiliac, spine
Gotuzzo et al., 1987	Peru	N/A	N/A	92	22	0.24	Sacroiliac, knee, ankle, spine
Guler et al., 2014	Turkey	3–82	N/A	370	178	0.48	Sacroiliac, spine, bursa
Gur et al., 2003	Turkey		N/A	283	195	0.69	Spine, sacroiliac
Hizel et al., 2007	Turkey	15–81	N/A	163	72	0.44	Spine, sacroiliac, paravertebral
Issa and Jamal, 1999	Jordan	3–14	N/A	68	38	0.56	N/A
Jia et al., 2017	China	3–75	N/A	590	137	0.23	Sacroiliac, knee, spine
Kazak et al., 2016	Turkey	15–85	N/A	164	87	0.53	Sacroiliac, hip, ankle, knee, spine
Khateeb et al., 1990	Kuwait	13–75	N/A	400	104	0.46	Sacroiliac, hip, knee, spine
Kokoglu et al., 2006	Turkey	15–69	67/71 (48.5/51.5%)	138	64	0.14	Sacroiliac, spine, peripheral arthritis
Kose et al., 2014	Turkey	14–83	N/A	72	10	0.31	Sacroiliac, spine
Kouba et al., 2013	Tunisia	19–74	23/9 (72/28%)	146	32	0.22	Spine
Kursun et al, 2013	Turkey	N/A	N/A	447	137	0.31	Spine
Lulu et al., 1988	Kuwait	10–60	N/A	400	105	0.26	Sacroiliac, spine, hip, knee, shoulder, ankle, elbow
Memish et al., 2000	Saudi Arabia	0–40	N/A	160	68	0.42	Sacroiliac, spine, hip, knee,ankle
Memut et al., 2012	Turkey	15–77	N/A	231	70	0.37	Sacroiliac, spine, bursa
Mugahi et al., 2014	Iran	11–80	N/A	81	8	0.099	N/A
Namiduru et al., 2003	Turkey	16–70	7/7/ (50/50%)	186	14	0.08	Spine
Okur et al., 2012	Turkey	2–16	N/A	147	20	0.14	N/A
Parlak et al., 2015	Turkey	1–16	N/A	496	55	0.11	Peripehral arthritis
Pourbagher et al., 2006	Turkey	2–77	N/A	251	114	0.45	Sacroiliac, spine, hip, bursa
Qehaja-Bucaj et al., 2015	Kosovo	2–74	N/A	124	55	0.44	Sacroiliac, spine, hip
Roushan et al., 2004	Iran	16–90	N/A	469	69	0.15	Sacroiliac, spine, ankle, knee, hip, wrist, sternoclavicular
Roushan et al., 2005	Iran	3–15	N/A	111	35	0.32	Sacroiliac, spine, ankle, knee, hip, wrist, shoulder
Santiago et al., 2011	Portugal	N/A		90	44	0.49	N/A
Sasan et al., 2012	Iran	0–16	N/A	82	52	0.63	Knee and hip
Savas et al., 2007	Turkey	2–77	N/A	140	74	0.53	Sacroiliac, spine
Tasova et al., 1999	Turkey	15–75	51/36 (58.6/41.4%)	238	87	0.37	Sacroiliac, spine, knee, ankle, bursa
Ulug et al., 2011	Turkey	4–15	N/A	22	5	0.23	Sacroiliac, hip, spine, ankle, sternoclavicular
Zaks et al., 1995	Israel	N/A	N/A	90	40	0.41	Sacroiliac, spine, hip, knee, ankle, shoulder, elbow
Zamani et al., 2011	Iran	2–12	14/10 (58.3/41.7%)	96	24	0.25	Knee, hip, ankle, wrist elbow

For all individuals in the included studies, brucellosis was diagnosed based on the presence of inflammatory signs (pain, swelling, and tenderness) of the affected joints and one or more of other diagnostic methods including positive blood or synovial fluid culture; serology (using 2-mercapthoethanol-Standard Agglutination Test (1/160), *Brucella* Tube Agglutination Test (1:1280), *Brucella* Skin Test, Complement Fixation test, Rose Bengal test, Coomb’s test (1/320), Wright agglutination test, Immunofluorescence, or ELISA IgG and IgM); and anomalies of the bones and joints evident by varying imaging techniques. Participants in most of the included studies were diagnosed based on clinical signs and serology (70%), and only a few reported additional positive blood or synovial fluid culture (30%) (Table 2).

**Table 2:** Diagnostic methods of osteoarticular brucellosis used in included studies.

Study	Country (n)	Diagnostic methods			
		Inflammatory signs	Culture	Immunoassays	Radiographs
Aktug-Demir et al., 2014	Turkey (75)	Arthralgia	Blood culture	Standard tube agglutination test	Contrast enhanced MRI
Al-Eissa et al., 1990	Saudi Arabia (40)	N/A	Blood culture	*Brucella* microagglutination test	N/A
Ariza et al., 1993	Spain (62)	N/A	N/A	Standard tube agglutination test, Rose Bengal test, Coombs test	Plain radiographs, bone radionuclide scan
Aydoslu et al., 2006	Turkey (14)	N/A	Blood culture	N/A	N/A
Benjamin et al., 1992	Saudi Arabia (57)	N/A	Blood culture	N/A	N/A
Benjamin and Khan, 1994	Saudi Arabia (70)	Pain, swelling, redness, functional disability	Blood and synovial fluid culture	Standard tube agglutination test	Plain radiographs
Besharat et al., 2010	Iran (56)	Pain, fever, sweating	N/A	N/A	N/A
Bosilkovski et al., 2004a	Macedonia (196)	Pain, swelling, redness, functional disability	N/A	Standard tube agglutination test, *Brucella* Coombs test	Fabere test, plain radiograph, MRI, computed tomography, radionuclide bone scan, ultrasound
Bosilkovski et al., 2004b	Macedonia (162)	Pain, swelling, redness, functional disability	N/A	Standard tube agglutination test, *Brucella* Coombs test	Plain radiographs, bone radionuclide scan, ultrasound, MRI
Bosilkovski et al., 2010	Macedonia (299)	Pain, swelling, redness, functional disability	N/A	Standard tube agglutination test, *Brucella* Coombs test, Brucellacapt test	MRI, radionuclide bone scans, computerized tomography
Bosilkovski et al., 2013	Macedonia (133)	Pain, swelling, redness, functional disability	N/A	Standard tube agglutination test, *Brucella* Coombs test, Brucellacapt test	Stinchfield, Mennel test, abnormality on radiography, radionuclide bone scan or ultrasound examination, MRI, computed tomography
Bukharie, 2009	Saudi Arabia (54)	Fever, pain	Blood and bone marrow culture	Standard tube agglutination test, ELISA (IgM and IgG)	N/A
Bulut et al., 2011	Turkey (84)	Fever, sweating, arthralgia	Blood culture	Standard tube agglutination test	Plain radiographs, computed tomography scan, bone scan, MRI
Cirakli et al., 2015	Turkey (11)	N/A	Body fluid culture	Standard tube agglutination test	N/A
Colmenero et al., 1991	Spain (65)	Pain, swelling, redness, functional disability	N/A	Seroagglutination, Coombs, indirect immunofluorescence, Rose Bengal	Plain radiographs, radionuclide bone scan
Colmenero et al., 1992	Spain (58)	Pain, swelling, redness, functional disability	N/A	Wright, Coombs, rose bengal test, indirect immunofluorescence	Plain radiographs, bone radionuclide scan
Colmenero et al., 1996	Spain (2)	N/A	N/A	Wright, Coombs, indirect immunofluorescence	Computed tomography
Colmenero et al., 2008	Spain (96)	Pain, swelling, redness, functional disability	N/A	Standard tube agglutination test, Coombs antibrucella or immunocapture agglutination test	Computed tomography
Dabbagh and Rasool 2009	Iraq (45)	Pain, swelling, and restriction of movement	N/A	*Brucella* agglutination test and 2-Mercaptoethanol	Plain radiograph
Dahouk et al., 2005	Germany (11)	N/A	Blood culture	Standard tube agglutination test, ELISA IgM, IgG	N/A
Demiroglu et al., 2007	Turkey (51)	N/A	Bone marrow, sternoclavicular and psoas abscess culture	Standard tube agglutination test	N/A
Ebrahimpour et al., 2017	Iran (464)	Swelling, effusion, restriction of movement	N/A	Standard tube agglutination test and 2-Mercaptoethanol	Plain radiographs, MRI, sonography
Eini et al., 2012	Iran (118)	Arthralgia	Blood culture	Standard tube agglutination test, *Brucella* Coombs test, 2-Mercaptoethanol	N/A
Fanni et al., 2013	Iran (26)	N/A	Blood and bone marrow culture	Serum agglutination test, Wright and Coombs test, 2-Mercaptoethanol	N/A
Fruchtman et al., 2015	Israel (92)	Fever, myalgia, headache	Blood culture	Standard tube agglutination test	N/A
Gonen et al., 2013	Turkey (50)		Blood and synovial fluid and bone marrow culture	Standard tube agglutination test, Coombs test	Plain radiographs, MRI, computed tomography, ultrasonography
Gotuzzo et al., 1987	Peru (22)	N/A	Blood, bone marrow, synovial culture	N/A	N/A
Guler et al., 2014	Turkey (178)	Pain, swelling, redness, functional disability	Blood culture	Standard tube agglutination test	Radiological alterations and/or radionuclide uptake in any deep joint
Gur et al., 2003	Turkey (195)	Pain, swelling, redness, functional disability	Blood and body fluid culture	Standard tube agglutination test	Plain radiographs, bone radionuclide scan, computed tomography, MRI
Hizel et al., 2007	Turkey (72)	Pain, fever	Blood culture	Standard tube agglutination test	Bone Scintigrapghy
Issa and Jamal, 1999	Jordan (38)	Arthralgia	Blood and bone marrow culture	Rose Bengal test, ELISA IgM and IgG, Wright test	N/A
Jia et al., 2017	China (137)	Fatigue, fever, muscle and joint pain, headache	Blood culture	Standard tube agglutination test	N/A
Kazak et al., 2016	Turkey (87)	Arthralgia	Blood and body fluid culture	Standard tube agglutination test and Rose bengal test,	N/A
Khateeb et al., 1990	Kuwait (104)	Pain, swelling, restriction of movement	Culture negative	Microagglutination, slide agglutination test, ELISA (IgG, IgM, and IgA)	Plain radiographs- no apparent pathological changes
Kokoglu et al., 2006	Turkey (64)	N/A	Blood and body fluids culture	Wright agglutination test	N/A
Kose et al., 2014	Turkey (10)	Arthralgia	Blood and bone marrow culture	Standard tube agglutination test	N/A
Kouba et al., 2013	Tunisia (32)	Pain	Blood and body fluids/tissue culture	Standard tube agglutination test	MRI, computed tomography
Kursun et al, 2013	Turkey (137)	Arthralgia	Blood and bone marrow culture	Standard tube agglutination test	Whole-body bone scintigraphy (Technetium 99m)
Lulu et al., 1988	Kuwait (105)	Pain and swelling	Culture negative	Microagglutination, slide agglutination test, ELISA (IgG, IgM, and IgA)	N/A
Memish et al., 2000	Saudi Arabia (68)	N/A	Blood and body fluid culture	Microagglutination test	N/A
Memut et al., 2012	Turkey (70)	Pain, swelling, redness, functional disability	Blood and bone marrow culture	Standard tube agglutination test, Rose Bengal test	Computed tomography, MRI
Mugahi et al., 2014	Iran (8)	Arthralgia	N/A	Wright test and 2-Mecarptoethanol test	N/A
Namiduru et al., 2003	Turkey (14)	Arthralgia	Blood and body fluid culture	Wright test	Plain radiograph, bone scintigraphy, MRI
Okur et al., 2012	Turkey (20)	Arthralgia	Blood culture	Standard tube agglutination test	N/A
Parlak et al., 2015	Turkey (55)	N/A	Blood culture	*Brucella* agglutination test, standard tube agglutination test	None
Pourbagher et al., 2006	Turkey (114)	Arthralgia	Blood culture	Standard tube agglutination test	Joint sonography, radiography, radionuclide, bone scintigraphy, and MRI
Qehaja-Bucaj et al., 2015	Kosovo (55)	Fever, arhtralgia, fatigue, sweating	N/A	Wright test	Plain radiographs
Roushan et al., 2004	Iran (69)	Pain, swelling, redness, functional disability	N/A	Standard tube agglutination test, 2-Mercaptoethanol	Plain radiographs, bone radionuclide scan
Roushan et al., 2005	Iran (35)	Pain, swelling, redness, functional disability	Blood culture	Standard tube agglutination test, 2-Mercaptoethanol	Radionuclide scan
Santiago et al., 2011	Portugal (44)	N/A			N/A
Sasan et al., 2012	Iran (52)	Arthralgia	Blood, urine and joint fluid culture	Wright test, Coombs test, Rose Bengal test, and 2-Mercaptoethanol	N/A
Savas et al., 2007	Turkey (74)	Arthralgia	Blood culture	Standard tube agglutination test	N/A
Tasova et al., 1999	Turkey (87)	Pain, swelling, redness, functional disability	Blood and body fluid culture	Standard tube agglutination test	Plain radiograph, radionuclide bone scan
Ulug et al., 2011	Turkey (5)	Pain, swelling, redness, functional disability	N/A	Standard tube agglutination test	Computed tomography, MRI, plain Radiograph
Zaks et al., 1995	Israel (40)	Pain, swelling, redness, functional disability	Positive culture	Standard tube agglutination test, Rose Bengal test, 2-Mercaptoethanol	Plain radiographs
Zamani et al., 2011	Iran (24)	Pain, swelling, redness, functional disability	Blood culture	Standard tube agglutination test, 2-Mercaptoethanol	N/A

### Prevalence

The prevalence estimates ranged from 27% in low-risk regions (e.g. Europe and North America) to 36% in high-risk regions (e.g. Middle East and South America), with no significant difference between the two estimates, indicating that the prevalence of OAB is independent of the endemicity of a particular region. High-risk regions included those countries where high number of cases or incidence of brucellosis have been consistently reported, such as in the Middle East, Asia, South and Central America, and Africa [27–29,32–36].

### Meta-analysis

**Fig 2:**
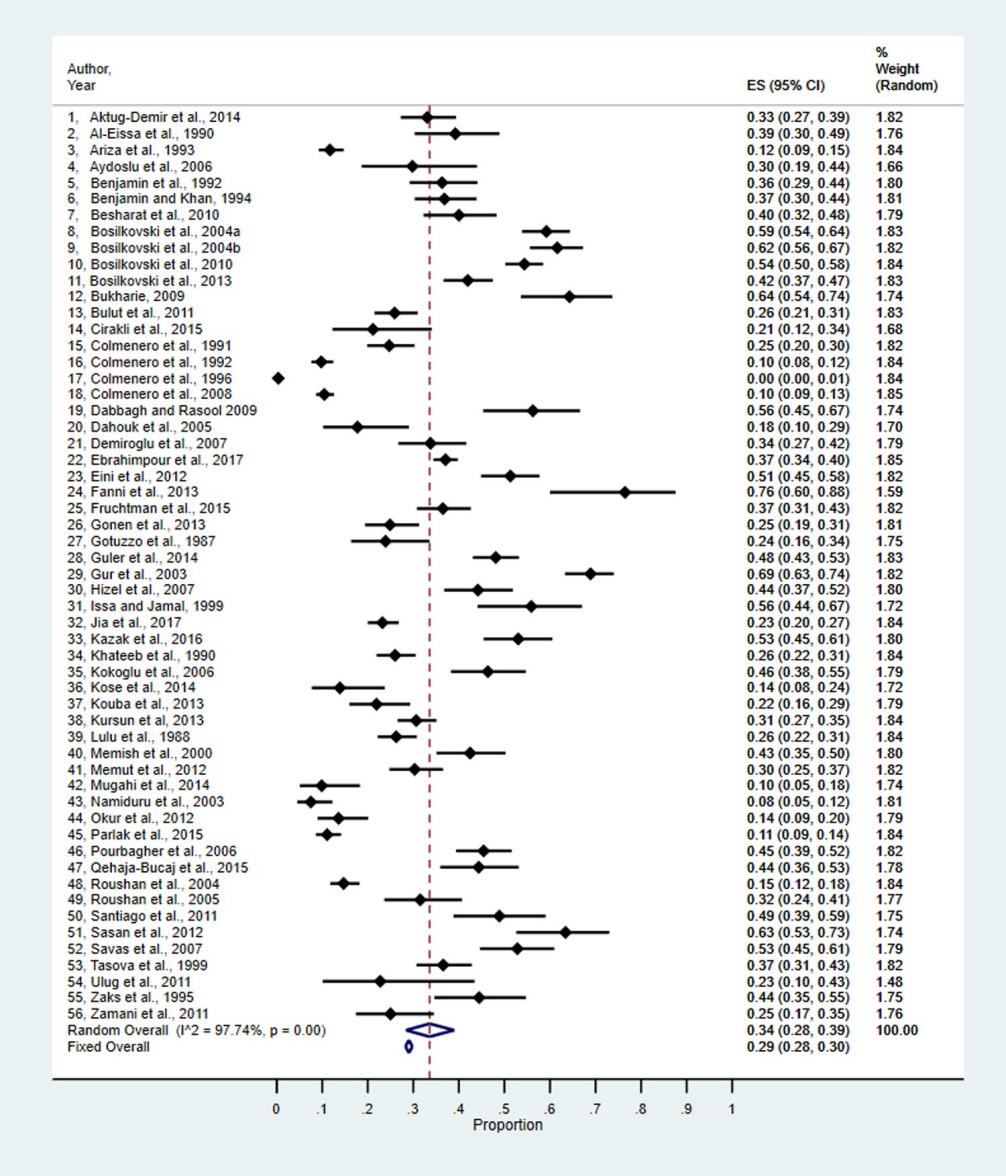
Metaprop results of prevalence estimates of osteoarticular brucellosis.

Fig 2 reflects the “metaprop” results of 56 prevalence estimates (converted back from the Freeman-Tukey transformations). The overall fixed effect estimate of prevalence was 0.29 (95%CI: 0.28 to 0.30). The fixed effect estimate was statistically heterogeneous with an I^2^ of 98.66%. The random effects estimate was 0.34 (95%CI: 0.28 to 0.39).

**Fig 3:**
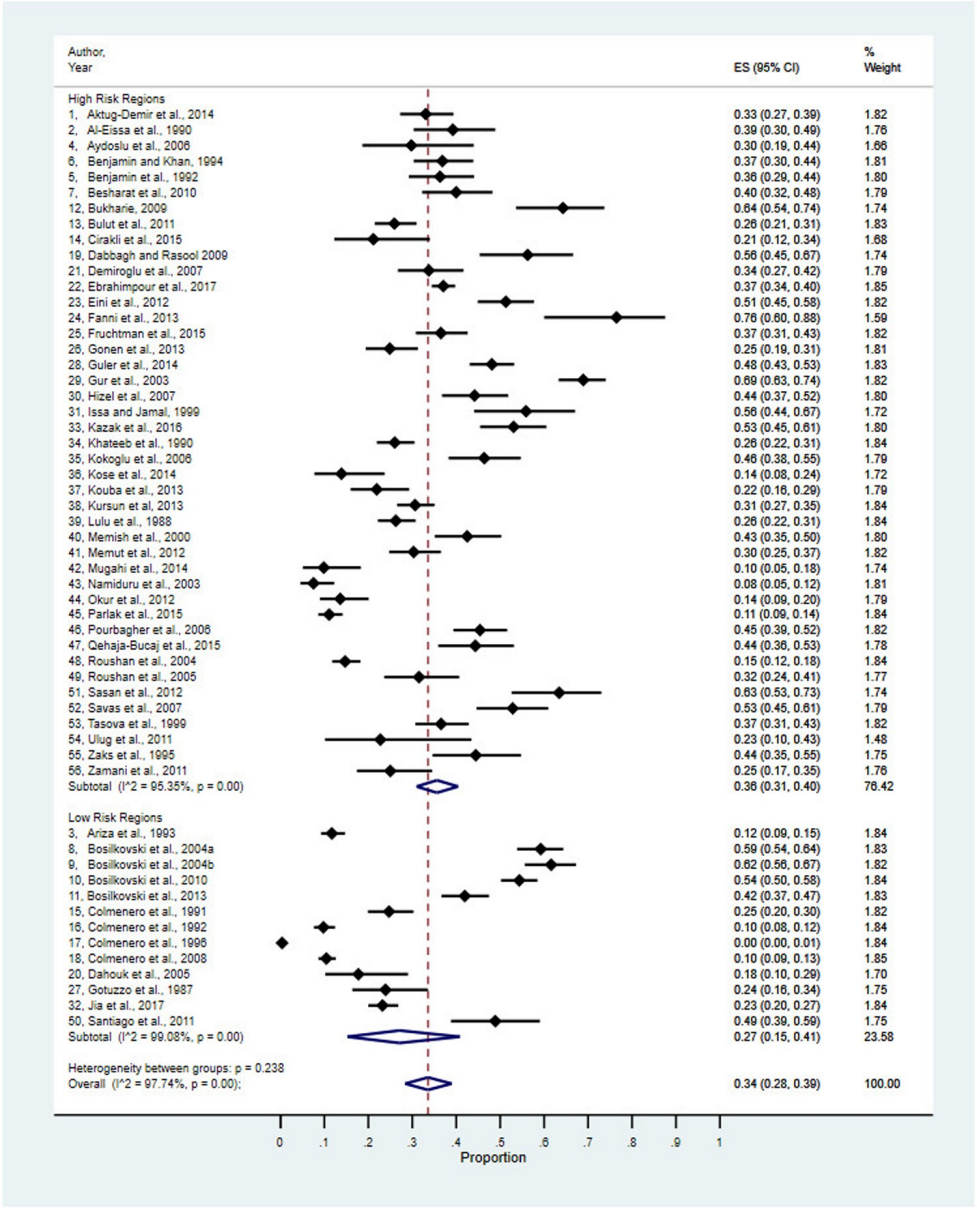
Prevalence estimates stratified by risk regions.

Fig 3 reflects the 56 prevalence estimates stratified by risk regions, which was determined based on previous reports of a high brucellosis incidence [27–29,32–36].

Both subgroups (high-risk and low-risk regions), as well as the overall result (Fig 3) were statistically heterogeneous. Stratification by risk regions alone was insufficient to explain the degree of heterogeneity. Random effects estimate for the two strata as prevalences were 0.36 (0.31 to 0.40) for high-risk countries and 0.27 (0.15 to 0.41) for low-risk countries. The estimates for the two strata were not statistically significantly different, suggesting that the prevalence of OAB is independent of the exposure risk of a particular region.

Sources of heterogeneity between studies can also be explored using meta-regression. To determine the source of heterogeneity in these studies, as well as influence of age and gender on the prevalence of OAB, we collected data on age and gender. However, these data were problematic, and thus, meta-regression could not be used. The age of individuals was typically reported as a range, for example, 16 to 75 years of age. Mean and/or median age of the population would have been more desirable for a meta-regression analysis. Additionally, some studies did not report any age data. Therefore, these studies were dropped out of the meta-regression analysis. Studies that did not report gender proportions were also excluded, and thus, meta-regression could not be used to further explore heterogeneity based on both age and gender.

**Fig 4:**
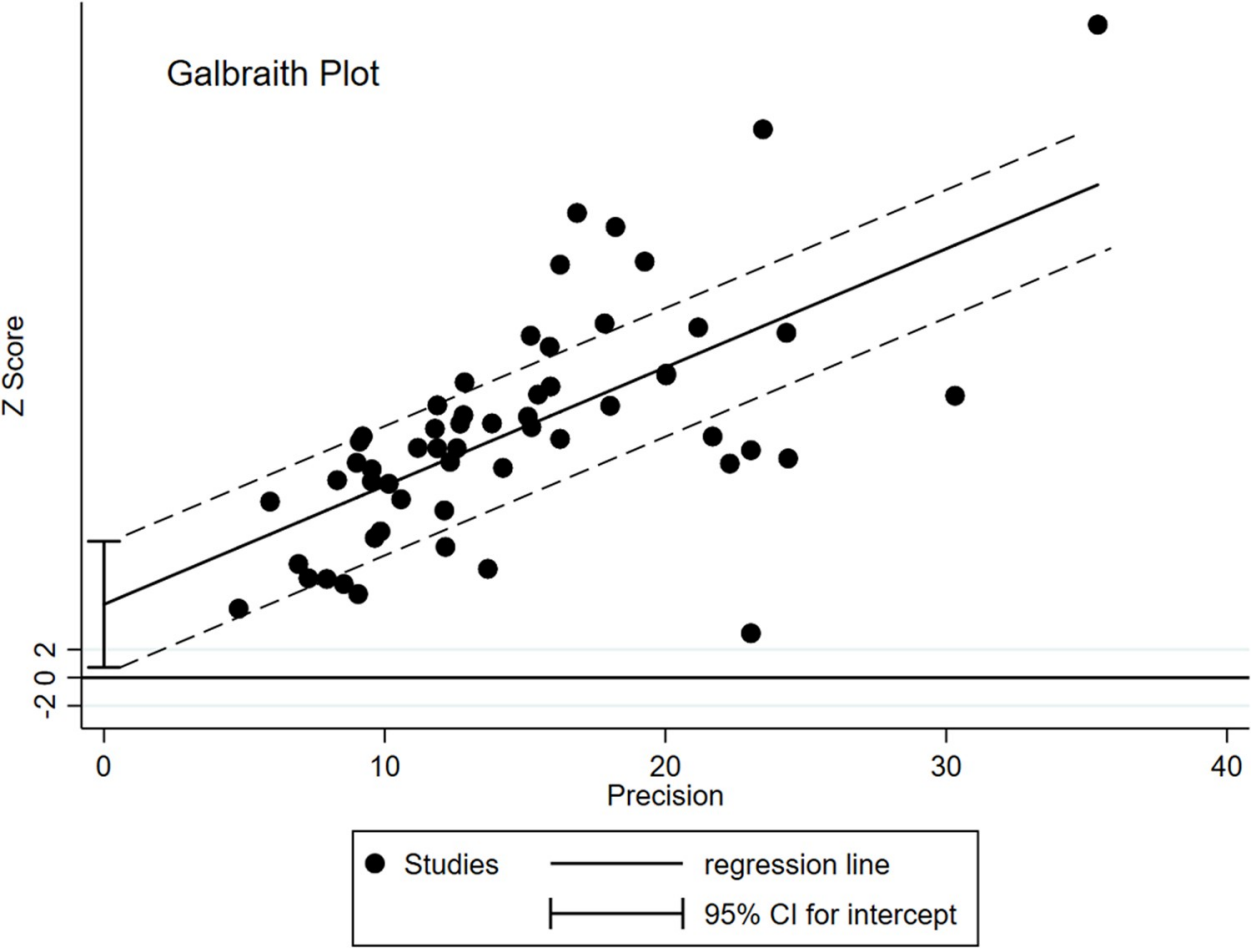
Galbraith plot analysis of all the included studies.

Outlier analysis has also been used to explain, and by removal of studies one at a time, explain heterogeneity. The degree of heterogeneity was such that outlier study removal would have left too few studies to calculate reasonable estimates. Galbraith plot is provided (Fig 4) using the Stata command “galbr” and the Excel computation of the Freeman-Tukey double arcsine transformation of prevalence values. Outlier studies are recognized as those outside the 2 parallel galbraith bands at values 2 and -2.

## Discussion

The main objective of this study was to systematically review the literature and perform a meta-analysis to estimate the global prevalence of osteoarticular brucellosis (OAB). A total of 56 studies met the inclusion criteria and were included in the systematic review and meta-analysis. Although there was an evidence of geographical variation in the prevalence of OAB with estimates ranging from 27% in low-risk regions to 36% in high-risk regions, the difference was not significant. This result indicates that the prevalence of OAB is not dependent on the endemicity of brucellosis in a region, and that brucellosis patients have at least a 27% chance of developing osteoarticular disease. In addition, the result also suggests that brucellosis remains an important public health concern in both high-risk [74,75], and low-risk regions [42,43,76]. Therefore, early pathogen detection using sensitive and specific validated diagnostic techniques as well as treatment of the disease to stop disease manifestation are paramount in the control of OAB.

Classification of a region into high or low-risk was determined based on previous reports of consistently high incidence of brucellosis in different countries in Africa, Asia, Eastern Europe, Mediterranean Basin, Middle East, South and Central America, and The Caribbean, as signified by the Centers for Disease Control and Prevention [27–29,32–36]. Low-risk regions include those countries with little or no reports of incidence of brucellosis such as North and South America, and some parts of Europe. In low-risk regions, very few occasional cases of brucellosis occur, and are usually travel-related. For example, consumption of raw animal products (e.g. raw milk or cheese) while visiting high-risk countries [77]. Furthermore, OAB may be difficult to diagnose in low-risk regions, not due to a lack of appropriate infrastructure, but because the disease is less common and may be confused with other causes of arthritis in humans (e.g. Lyme disease), hence, an under-diagnosis or misdiagnosis of the disease.

In this current study, although not statistically significant, the higher prevalence of OAB in high-risk regions correlates with previous findings reporting a high incidence of brucellosis in Middle Eastern countries such as Iran and Saudi Arabia [75,78,79]. Moreover, prevalence variation in different parts of the world may be due to varying environmental and socioeconomic factors such as sanitation, availability of medical facilities for optimum treatment and care, brucellosis awareness in communities, diagnostic capabilities for prompt detection of the disease condition, amongst others [80–83]. Another important factor is under-diagnosis or misdiagnosis of brucellosis because the disease manifests as flu-like symptoms and may bear resemblance to other diseases with similar symptoms such as malaria, or dengue fever, which are common disease conditions in many parts of Africa, thus leading to delays in detection and appropriate treatment of the disease [81,82]. Also, *Brucella*-induced arthritis in older individuals is commonly overlooked as arthritis due to old age. Hence, an understanding of patients’ history and thorough clinical examinations are recommended.

Additionally, higher prevalence in high-risk regions may be due to close interactions with domestic animals such as raising animals in close proximity to human living areas, low public awareness of brucellosis as a serious debilitating disease, resistance to slaughtering infected animals, and the customary beliefs of raw milk ingestion [79,84].

Focal complications of brucellosis, such as osteoarticular involvement are frequently reported in children, especially in high-risk regions. For example, childhood OAB (age ≤ 18years) was reported in 25% of the studies included in this current meta-analysis study [85–91], while 8% of the studies reported OAB in adults only.

Clinical presentation of childhood brucellosis is similar to those observed in other flu-like illness such as malaria, influenza, or dengue and is often misdiagnosed and mistreated, especially in resource-limited settings [13,81,82,92–94]. In most reported cases, contact with contaminated animal products or consumption of raw unpasteurized milk has been shown as a risk factor for contracting the disease. For example, in some resource-limited settings, the available milk is rather given to children than adults, and if the milk is contaminated, it poses an increased risk of brucellosis infection. Most commonly, *B*. *melitensis* is the main causative agent in infected children, although other species such as *B*. *abortus* and *B*. *suis* have also been identified [13,36,38,91].

Most studies in this review reported age range for individuals presenting OAB, for example, an age range of 16–75 years, while some studies did not report any age data. Therefore, it was impossible to determine the actual variation in prevalence based on age of the study population. Thus meta-regression could not be used to further explore heterogeneity.

As regards gender, both sexes are affected equally, although some reports claim that the disease is more prevalent in males (80%) than in females (19%) because of the nature of the male job in such regions, which facilitates increased exposure to animals and their products, as observed in herdsmen, ranchers, pastoralists and abattoir workers [29,38,51]. In this current study, because of the limited information provided in the selected articles, it was impossible to determine variation in OAB prevalence based on gender of the study population.

There are several diagnostic tools for brucellosis. The gold standard of brucellosis diagnosis is the positive culture of *Brucella* from tissues and bodily fluids (e.g. blood, bone marrow, synovial, and cerebrospinal fluid) of infected patients, although culture yield is inversely related to the duration of illness [95–97]. For example, culture yield is greater during the acute stage of brucellosis while it is less in later stages of the disease or during occasional relapses [84]. Additionally, the likelihood of isolation in patients with chronic disease and focal complications can be improved by using sampling material from affected sites, such as synovial fluid in OAB cases [97].

Various *Brucella* culture systems including automated continuously monitored blood culture systems such as Bactec (BD Diagnostics, Sparks, MD, USA) and BacTAlert (bioMerieux, Durham, NC, USA) give higher yields than the conventional culture method and facilitate the detection of bacterial growth [84,98]. However, these culture systems are not routinely used in most high-risk regions because of insufficient infrastructure as well as trained personnel. Hence, classical bacteriological culture is a common diagnostic method for brucellosis in these regions because it is easily accessible [95–97].

Due to inconsistent yield of *Brucella* from culture systems, increased risk of personnel infection, as well as the lack of validated molecular-based diagnostic techniques, the common standard for diagnosis of brucellosis is serological assays, which include Serum Agglutination Test (SAT), Microagglutination Test (MAT), Enzyme Linked Immunosorbent Assay (ELISA), Indirect Coombs (Anti-Human Globulin) Test, Brucellacapt, Wright agglutination test, Rose Bengal Slide Agglutination Test (RB-SAT), Complement Fixation Test (CFT), Indirect immunofluorescence test (IF), and Immunochromatography Lateral Flow Assay. Among the serological assays, SAT is the most frequently used and standardized test. SAT is based on measuring an agglutination titer of different serum dilutions (1:20–1:1280) against a standardized concentration of whole *Brucella* cell suspension. The highest serum dilution showing more than 50% agglutination is considered the agglutination titre. A positive titre is 1:160 or more [84,98,99]. Multiple testing at 4–8 week intervals is recommended to overcome the drawback of inconsistent results. ELISA is another commonly used serological assay for diagnosing brucellosis. It is considered specific (95%) and sensitive (98%) and has been consistently shown to diagnose both focal and chronic brucellosis [98,100]. Generally, because of the variability in the specificity and sensitivity of the conventional serological tests routinely used in high-risk regions, a combination of varying serological tests (e.g. SAT and either indirect Coombs, Brucellacapt, or ELISA for IgG and IgM) is recommended for the definitive diagnosis of human brucellosis [97,98,100–102].

Correspondingly, all studies included in this systematic review and meta-analysis used a combination of serological tests including SAT (1/160), ELISA (IgG and IM), CFT, IF, Wright agglutination test, *Brucella* Tube Agglutination Test (1:1280), *Brucella* Skin Test, Coomb test (1/320), or RB-SAT (Table 2 shows the diagnostic tests used by the respective studies). All individuals presenting OAB in the articles selected for the current analyses were positive for two or more of the reported diagnostic methods (such as clinical signs of fever, inflamed joints, myalgia, arthralgia, and/or bacteriological culture, and serology) Table 2.

In addition to clinical and serological diagnosis of OAB, radiographic abnormalities of the bones and joints, which manifest as arthritis, sacroiliitis, and spondylitis, have been described using varying radiological techniques such as radionuclide bone scan, plain radiography, joint sonography, computed tomography, and contrast-enhanced magnetic resonance imaging, amongst others. The abnormalities in the affected osteoarticular regions included joint space narrowing or widening, subchondral erosion, subchondral sclerosis and/or soft tissue swelling [37,46,63,103]. For example, bone scans were considered positive for abnormalities when there was increased uptake of the compound in the respective osteoarticular regions [46]. Generally, radiological diagnosis of OAB in humans is nonspecific and inconsistent, but varying degrees of abnormalities of affected regions have been described [46,63]. In this current study, most of the individuals had a report of varying bone abnormalities evident by the respective imaging techniques.

Overall, the prospects of OAB diagnosis by a physician in high-risk versus low-risk regions differ. *Brucella*-induced osteoarticular involvement can be easily suspected in high-risk regions based on clinical signs and history of contact with animals and raw animal products, thereby facilitating rapid diagnosis and treatment. However, in low-risk regions, especially where brucellosis has been eradicated, a knowledge of patients’ clinical history (e.g. a travel-related exposure to and consumption of raw animal products such as milk and cheese) is particularly valuable to the diagnosis of OAB (13).

Since the clinical features of OAB are not specific and there is yet to be a single consistent definitive diagnostic technique, the clinical history of animal contact or consumption of raw animal products is especially important, as well as a combination of diagnostic methods (bacteriological culture, serology and imaging).

The purpose of the current study was to estimate the prevalence of OAB among brucellosis patients worldwide by performing a meta-analysis. For the first time, we have demonstrated that the prevalence of OAB is independent of brucellosis endemicity of a particular region, and that brucellosis patients have at least a 27% chance of developing an osteoarticular disease. Thus, brucellosis remains a public health concern in both high-risk and low-risk countries [104], although there are some limitations to this current study, such as incomplete data representation. For example, lack of vital demographics precluded the feasibility of estimating OAB prevalence based on age and gender. Nevertheless, the current review is still very valuable, and has contributed to our understanding of the global prevalence of *Brucella*-induced osteoarticular disease. Hence, this study has provided the basis for increased awareness of OAB, the need for the development and validation of diagnostic tests, and appropriate treatment regimen to reduce disease manifestation. Therefore, further research should investigate the potential mechanisms of OAB, as well as the influence of age, gender, and other socioeconomic factor variations in its global prevalence, as this may provide insight into associated exposure risks and management of the disease.

## Supporting information

S1 Table PRISMA checklist. (DOCX)

S1 Text Medline search. (DOCX)
