# Supplementary material for: A systematic review and meta-analysis of the prevalence of osteoarticular brucellosis
Source: PLoS Negl Trop Dis. 2019 Jan 18;13(1):e0007112. doi: 10.1371/journal.pntd.0007112 (PMC6355028; doi:10.1371/journal.pntd.0007112)
Supplement: S1 Text — (DOCX) [file pntd.0007112.s002.docx]

S1 Text: Medline (Ovid) search

1. exp Brucellosis/ or (Brucellosis or brucella).ti,ab,kw.

2. ((bone or joint) adj1 (disease* or infection*)).ti,ab,kw.

3. exp Arthritis, Infectious/ or exp Osteomyelitis/

4. exp Spondylitis/

5. bone diseases/ or exp joint diseases/ or exp arthralgia/ or exp arthritis, experimental/ or exp arthritis, infectious/ or exp osteoarthritis/ or exp sacroiliitis/ or exp spondylarthritis/ or exp bursitis/

6. (Arthritis or osteoarthritis or sacroiliitis or Spondylarthritis or bursitis or arthralgia or Osteomyelitis or Spondylitis).ti,ab,kw.

7. or/2-6

8. 1 and 7

9. exp Prevalence/

10. exp Incidence/

11. exp Morbidity/

12. (prevalence or incidence or morbidity or epidemiolog*).ti,ab,kw.

13. exp Epidemiologic Studies/

14. or/9-13

15. 8 and 14
